# Efficacy of atypical antipsychotics in schizophrenia patients: effects of *5-HTR* SNPs

**DOI:** 10.1186/s12991-025-00547-z

**Published:** 2025-02-18

**Authors:** Keying Liu, Bide Zhang, Zhoufangyuan Chen, Fukun Chen, Zexu Li, Yunzhi Gao, Yuechao Zhao, Yihao Liu, Yanlong Wang

**Affiliations:** 1 Lin He’s Academician Workstation of New Medicine and Clinical Translation in Jining Medical University, Jining Medical University, Jining, China; 2 College of Basic Medicine, Jining Medical University, Jining, China

**Keywords:** *5-HTR*, SNPs, Atypical antipsychotics, Schizophrenia, Clinical efficacy

## Abstract

The 5-hydroxytryptamine receptor (5-HTR) is a key protein responsible for the effects of 5-hydroxytryptamine (5-HT) and an important target for many antipsychotics. *5-HTR* has a high degree of genetic polymorphism, and atypical antipsychotics are 5-HTR antagonists widely used in treating schizophrenia. With the increasing development of medical technology, antipsychotics are being updated rapidly, and their efficacy and safety are being optimised. However, owing to the complexity of patients’ genetic polymorphisms and psychiatric disorders, there are still individual differences in clinical efficacy. This article reviews the typing of 5-HTR, a common target of clinical atypical antipsychotics, and the effects of *5-HTR* gene single nucleotide polymorphisms (SNPs) on the efficacy of atypical antipsychotics. Specific genotypes of six types of *5-HTR* genes are associated with differential responses to atypical antipsychotics, which may help guide the development of individualized clinical treatments for patients with schizophrenia.

## Introduction

Schizophrenia is a genetically influenced and phenotypically diverse clinical disorder that can severely affect patients and society. Atypical antipsychotics are the first treatment choice for acute symptoms and chronic schizophrenia [1]. However, some patients experience only partially effective or ineffective symptoms [2]. Thus, there are significant individualised differences in the response of patients with schizophrenia to antipsychotic treatments. 5-Hydroxytryptamine (5-HT), also known as serotonin because it was first discovered in serum, is an autoactive substance widely found in the mammalian digestive system, blood platelets, cortical layers of the brain, and nerve synapses [3]. 5-HT in the brain regulates pain, sleep, body temperature, sexual behaviour, and mental mood, but only when it binds to the 5-HT receptor (5-HTR). Epidemiological studies have shown significant genetic heterogeneity in 5-HT-related gene function and in vivo effector systems in patients with psychiatric disorders [3–5]. Notably, most currently available antipsychotics act by modulating 5-HT levels in vivo, and the regulation of different 5-HTR variations can mediate the production of different 5-HT bioactivity at different sites [6].

Seven types of 5-HTR have been identified and can be further categorised into several subtypes, and different types of 5-HTR play different roles in the human body. 5-HTR, distributed throughout the human brain, regulates various psychiatric behavioural activities [7]. Atypical antipsychotics, in partticular, act as partial agonists at 5-HTR1A and as antagonists at 5-HTR2A [8]. This dual action is crucial for their therapeutic efficacy. Morver, research has suggested that the clinical response to atypical antipsychotics may be a function of single nucleotide polymorphisms (SNPs) including *5-HTR* genes [9]. Thus, *5-HTR* SNPs are associated with atypical antipsychotic efficacy. This article outlines the *5-HTR* SNPs and their effects on atypical antipsychotic efficacy, providing evidence-based medical evidence for the application of antipsychotic drugs in psychiatry.

### Types of 5-HTR

5-HTR is categorised into seven types: 5-HTR1, 5-HTR2, 5-HTR3, 5-HTR4, 5-HTR5, 5-HTR6, and 5-HTR7, which can be further categorised into several subtypes [10]. 5-HTR1 is the most genetically diverse type and has five subtypes: 5-HTR1A, 5-HTR1B, 5-HTR1D, 5-HTR1E, and 5-HTR1F [11]. The 5-HTR2 type has three subtypes: 5-HTR2A, 5-HTR2B, and 5-HTR2C (previously known as 5-HTR1C) [12]. The 5-HTR3 type has five subtypes: 5-HTR3A, 5-HTR3B, 5-HTR3C, 5-HTR3D, and 5-HTR3E [13]. 5-HTR5 has two subtypes: 5-HTR5A and 5-HTR5B [14]. No related subtypes have been found for 5-HTR4, 5-HTR6, or 5-HTR7. Among them, 5-HTR1D can be further categorised into 5-HTR1Dα and 5-HTR1Dβ. As 5-HTR1Dβ is completely homologous to 5-HTR1B, 5-HTR1D refers to 5-HTR1Dα.

### Clinical application of atypical antipsychotics

Atypical antipsychotics have been used in clinical practice since the 1990s and have become the first-choice medication for treating schizophrenia. They also play a significant role in mood stabilisation for depression, bipolar disorder, and mania. Most of these drugs share similar properties, and most neuropharmacologists agree that atypical antipsychotics must possess three key characteristics. Firstly, they should demonstrate better efficacy in treating schizophrenia. Secondly, they should have fewer or no extrapyramidal reactions. And finally, they shuold have dual-blocking effects on 5-HT and dopamine D2 receptors [15]. The primary pharmacological difference between atypical and classical antipsychotics lies in their higher binding affinity for 5-HTR2A and dopamine D2 receptors [16]. Clinical trials have shown that an increasing number of patients with schizophrenia exhibit good compliance and tolerance to atypical antipsychotics, resulting in reduced relapse rates to some extent [17].

Atypical antipsychotics relieve positive and negative symptoms in patients with schizophrenia by primarily blocking dopamine receptors and 5-HTR. Notably, in their research, Kapur et al. recommended that atypical antipsychotics bind to dopamine D2 receptors and rapidly disengage within a short period to effectively treat schizophrenia, as prolonged binding can cause extrapyramidal reactions [18].

Atypical antipsychotics can bind to various receptors, including 5-HTR, dopamine receptors, α1 and α2, H1, and M1 receptors. Notably, binding to 5-HTR2A/D2 receptors improves positive and negative symptoms, binding to 5-HTR2C/D2 receptors improves affective and cognitive impairment, binding to 5-HTR1A/D2 receptors improves negative symptoms and reduces dyskinesia, and binding to 5-HTR1D inhibits 5-HT release and exhibits anxiolytic and depressant properties [19]. Moreover, by blocking D2 receptors, the function of dopamine is reduced, leading to a decrease in extrapyramidal responses [20].

Atypical antipsychotics, such as olanzapine, clozapine, risperidone, aripiprazole, and lurasidone, have several advantages over classical antipsychotics, including broader action, better efficacy, and improved safety profiles. Among these, when considering Chinese patients with first-episode schizophrenia, risperidone may be a better choice than aripiprazole due to its superior efficacy and patient tolerance [21]. However, aripiprazole is preferred if short-term weight gain is to be avoided, whereas olanzapine is necessary to prevent neurological adverse effects (dystonia, rigidity, hypokinesia/akinesia, hyperkinesia, tremor, akathisia, epileptic seizures and paraesthesia) [21]. In contrast, in Indian patients with schizophrenia, olanzapine is more effective than risperidone in improving negative symptoms, while risperidone carries the risk of causing hyperprolactinemia [22]. Furthermore, clozapine has demonstrated positive efficacy in refractory schizophrenia and reduced suicide rates [23].

Risperidone has a high affinity for 5-HTR2, 5-HTR6, and 5-HTR7 [24]. Research has shown that it poses a lower risk of relapse in adult US outpatients with clinically stable schizophrenia or schizoaffective disorder compared to haloperidol [25]. Aripiprazole, on the other hand, binds more uniquely to receptors, acting as a partial agonist at both the D2 receptor and 5-HTR12A, as well as a partial agonist at 5-HTR1A [26]. Furthermore, clinical studies have proven that aripiprazole is more effective than a placebo in treating acute schizophrenia relapse, chronic schizophrenia, and acute mania [27].

### Association between single nucleotide polymorphisms of the *5-HTR* gene and the efficacy of atypical antipsychotics

#### Literature retrieval strategy

The English literatures that examined the relationship between 5-HTR gene SNPs and atypical antipsychotics drug efficacy were retrieved from PubMed, Embase, ScienceDirect, and Web of Science databases. The search terms included: “Schizophrenia”, “5-hydroxytryptamine receptor”, “HTR”, “5-HT”, “5-HTR”, “single nucleotide polymorphism”, “polymorphism”, “SNPs”, and “SNP”.

According to the method of our previously study [28], the included studies had to meet the following criteria: (a) the original data were in published studies that assessed the relationship between SNPs of the *5-HTR* gene and atypical antipsychotics drug efficacy; (b) the subjects of the original study were patients with schizophrenia; (c) during the experiment, all subjects took atypical antipsychotic drugs orally, and no longer received any other antipsychotic treatment; (d) in the original study, drug efficacy was evaluated by the Positive and negative symptom scale, Brief psychiatric rating scale, Clinical global impression, Global assessment scale, Scale for the assessment of negative symptoms, and prior criteria; a study was excluded if: (a) it was a review, a conference abstract, a commentary, a news reports or other similar type of publication; (b) it was a repeated publications; (c) it had incomplete or insufficient data; (d) it was irrelevant.

#### 5-HTR1A

A common *5-HTR1A* gene polymorphism is the rs6295 (−1019 C/G) polymorphism of the *5-HTR1A* gene, which is a functional SNP responsible for a C-G substitution at position 1019 in the promoter region [29]. This polymorphism has been found to regulate the gene’s transcription rate. Previously, research by Yoshikawa et al. demonstrated that rs6295 predicts the efficacy response to lurasidone in patients with schizophrenia of European ancestry, with patients carrying the G gene exhibiting reduced improvement in the acute phase [2]. In contrast, Bosia et al. discovered a significant effect of *5-HTR1A* on the Positive and Negative Syndrome Scale (PANSS) negative subscale, with greater improvement observed among *5-HTR1A* G/G subjects [29]. Additionally, the rs6295 polymorphism has been associated with anxiety and depression in those of Asians descent with G allele linked to improved efficacy [30].

Wang et al. revealed that risperidone is more effective at improving negative symptoms in Chinese schizophrenia patients with the CC genotype [31]. Similarly, a study by Reynolds et al. observed enhanced outcomes in Spanish Caucasian patients with first-episode schizophrenia who had the CC genotype (most of whom were treated with risperidone or olanzapine) [32]. However, the improvement mainly pertained to negative and depressive symptoms rather than positive symptoms [32]. Consequently, the rs6295 polymorphism could serve as a useful predictor for antipsychotic efficacy.

#### 5-HTR2A

##### rs6311 (−1438 G/A)

rs6311 is located 1438 bp upstream of the promoter region of 5-HTR2A, with allele A replaced with G. A study by Ellingrod et al. showed that olanzapine improved negative symptoms better in patients with genotype AA [33]. Similarly, a study by Yan et al. showed better outcomes in Han Chinese patients with acute paranoia who had the AA genotype [34]. Thus, the rs6311 polymorphism could be used to predict antipsychotic efficacy, and it may influence the efficacy of olanzapine. Additionally, Arranz et al. showed that among Caucasians of British origin treated with clozapine, patients with the GG genotype had significantly more non-responders than responders, In contrast, patients with the AA or AG genotypes demonstrated better efficacy [35, 36]. This suggests that rs6311 polymorphism may also influence the efficacy of clozapine.

##### rs6313 (T102C)

The rs6313 (T102C) polymorphism is located in exon 1 of the *5-HTR2A* gene, where the T allele is replaced by C. This polymorphism does not result in amino acid substitutions and is not considered a functional SNP [37]. Among patients with acute schizophrenia in an Italian Caucasian population, rs6313 was associated with different dimensions of symptoms for various antipsychotics. Specifically, it was linked to the disorganised thought dimension for risperidone and the depressive and anxiety dimensions for olanzapine. Notably, changes in the total PANSS score and various symptom dimensions (including the PANSS negative sub-score, PANSS positive sub-score, and Emsley’s positive factor [38]) after risperidone treatment were influenced by rs6313, with patients carrying the TT genotype showing a better response [39]. Conversely, patients with the TT genotype showed a poorer response to the Emsley anxiety factor after olanzapine treatment, while those with the CC genotype showed a poorer response to the Emsley positive factor [39].

These data further support the hypothesis that *5-HTR2A* affects antipsychotic efficacy. In a separate study of American Iowa schizophrenia patients treated with olanzapine, negative symptom reduction was associated with the TT genotype [40]. However, among Caucasian paranoid schizophrenia patients treated with olanzapine, those with the CC genotype demonstrated better improvements in positive PANSS scores [41].

Most studies concluded that olanzapine efficacy is higher in patients with the TT genotype. However, some studies also concluded that olanzapine efficacy was higher in patients with the CC genotype [42]. For instance, in Chinese Han in-patients with schizophrenia [42] and acute schizophrenia [43], those with the CC genotype responded better to risperidone (especially in terms of negative symptoms). Kim et al. also found that after treatment with risperidone, the C allele was more frequent in responders, while those with the TT genotype showed less clinical improvement than those with TC or CC genotypes [44]. Therefore, risperidone treatment maybe more effective in patients harbouring the C allele.

Regarding clozapine treatment, a study of Western European patients with schizophrenia found that non-responders were significantly more common among those with the CC genotype [45]. In contrast, patients with the TT and TC genotypes had a significantly higher rate of responders compared to non-responders [45], indicating that patients carrying the T allele demonstrated better efficacy. On the other hand, Shinkai et al. concluded that the T102C polymorphism was not associated with the pathogenesis or negative symptoms of schizophrenia [46]. Similarly, Kanno-Nozaki et al. found no association between the T102C polymorphism and the response to aripiprazole treatment [37]. Thus, rs6313 could serve as a potential marker for predicting the clinical efficacy of antipsychotics such as olanzapine, risperidone, and clozapine.

##### rs6314 (C1354T/His452Tyr/H452Y)

A study conducted by Blasi et al. demonstrated that rs6314 affects the expression of *5-HTR2A* and is linked to the effectiveness of olanzapine treatment [47]. Specifically, in Italian Puglial Caucasians treated with olanzapine, the T allele was associated with reduced improvements in negative symptoms and better efficacy in patients with the CC genotype [48]. Moreover, in Caucasian patients with paranoid schizophrenia, a superior response to olanzapine in patients with the His/His (CC) genotype was significantly associated with the percentage improvement in PANSS positive symptom scores [41]. Furthermore, Martín-Guerrero et al. discovered that the rs6314 polymorphism impacts the clozapine-induced phosphorylation-dependent signalling network, which subsequently affects the effectiveness of clozapine treatment [49].

Among Caucasians of British descent treated with clozapine, patients with the CT or CC genotypes demonstrated better efficacy [50]. Notably, the frequency of the Tyr452(T) allele was higher in non-responders compared to responders [35]. Additionally, in European Caucasians, there was an association between the Tyr452(T) allele and poor clinical responses to clozapine [51]. In contrast, in a study where 77.9% of the sample consisted of Caucasians with the His/His (CC) genotype, it was also observed that treatment with clozapine was more effective [52]. Therefore, patients with the CC genotype of rs6314 may experience improved efficacy with both olanzapine and clozapine. Additionally, Mata-Pastor et al. demonstrated that olanzapine and clozapine have similar effects on 5-HTR; however, further studies are necessary [53].

#### 5-HTR2C

The main SNPs associated with schizophrenia in the *5-HTR2C* gene are rs3813929 (-759 C/T) and rs518147 (-697G/C). In Chinese Han patients with first-episode schizophrenia treated with risperidone or chlorpromazine, the rs3813929 polymorphism was linked to negative symptoms and general psychopathology, and patients carrying the T allele responded poorly to antipsychotics, particularly male individuals [54]. In contrast, among Chinese Han patients treated with clozapine, those with the CC genotype demonstrated better efficacy [55]. Additionally, in female mainland Chinese Han patients treated with risperidone, three SNPs (rs518147, rs1023574, and rs9698290) were significantly associated with patient efficacy Overall, patients with the CC genotypes rs518147 and rs1023574 showed better efficacy, and patients with the TT genotype rs9698290 showed better efficacy [56].

#### 5-HTR3A

##### rs1062613 (C178T/Pro16Ser)

rs1062613 is located in the 5’-UTR region of *5-HTR3A* [57] and may affect 5-HTR3A expression. In Indian patients with refractory schizophrenia treated with clozapine, those with TT or TC genotypes showed better efficacy [58]. This finding is consistent with results from Italian patients, who also demonstrated improved efficacy with TT or TC genotypes [50]. Meanwhile, among Caucasians of German origin, patients with the TT or TC genotype also showed better efficacy (Study 1 used risperidone or haloperidol as the primary medication, while Study 2 used olanzapine as the primary medication and a variety of atypical antipsychotics, such as risperidone) [59]. However, one study also showed that patients with the CC genotype showed better efficacy than Caucasian patients treated with clozapine [60].

##### rs2276302

Among Indian patients with treatment-resistant schizophrenia who received clozapine, those with the GG or AG genotypes at the rs2276302 locus demonstrated superior effectiveness [58]. However, in Caucasian patients, those with the AA genotype exhibited better efficacy [60]. In the aforementioned study, the rs1062613 and rs2276302 polymorphisms accounted for only 13.8% of the variability in clozapine response, whereas combining clinical predictors explained 38% of the variability. These results indicate that the combination of clinical and pharmacogenetic models provides a stronger predictive value for drug efficacy [58].

For Caucasian patients with schizophrenia treated with clozapine, those with the AA genotype at rs2276302 and rs1062613 showed better efficacy [60]. These findings indicate a significant genotypic association between these two loci, suggesting that this *5-HTR3A* polymorphism may play a role in the efficacy of clozapine treatment [60]. In contrast, Gu et al. recommended that *5-HTR3A* polymorphisms may predict the response to risperidone treatment in Chinese patients with schizophrenia [61]. However, a study by Gutierrez et al. found similar allele and genotype distributions in clozapine responders and non-responders [62]. This study concluded that carriers of *5-HTR3A* and *5-HTR3B* are unlikely to exhibit different clinical efficacy regarding clozapine treatment. Therefore, the results of pharmacodynamic analyses differ among different studies.

#### 5-HTR6 and 5-HTR7

Among the rs6699866 polymorphisms of the *5-HTR6* gene, Chinese Han patients with schizophrenia who had the genotype AA or AT showed a better response to treatment with risperidone. Additionally, there was a more significant reduction in positive symptoms in the risperidone-treated group [63]. Furthermore, the rs1805054 (C267T) polymorphism has been shown to affect the response to risperidone in terms of positive symptoms and general psychopathology [42]. In one study, among Chinese Taiwanese Han patients treated with risperidone, those with the TT genotype demonstrated better efficacy [63]. They also exhibited a better treatment response to positive symptoms and general psychopathology compared to negative symptoms [24]. This was also seen in Chinese Han patients, including those admitted with acute schizophrenia [24]. However, Masellis et al. found no correlation between the rs1805054 polymorphism of *5-HTR6* and the response to clozapine treatment [64].

Regarding *5-HTR7*, Takekita et al. discovered that *5-HTR7* polymorphisms were not associated with an overall improvement in symptoms among Japanese patients with schizophrenia treated with either piperidone or aripiprazole [65]. Additionally, Wei et al. found no correlation between *5-HTR7* and antipsychotic efficacy, regardless of genotype or haplotype, among Chinese patients treated with risperidone [66]. Therefore, *5-HTR7* polymorphisms may not be reliable genetic markers for predicting the efficacy of atypical antipsychotics.

## Discussion

**Fig. 1:**
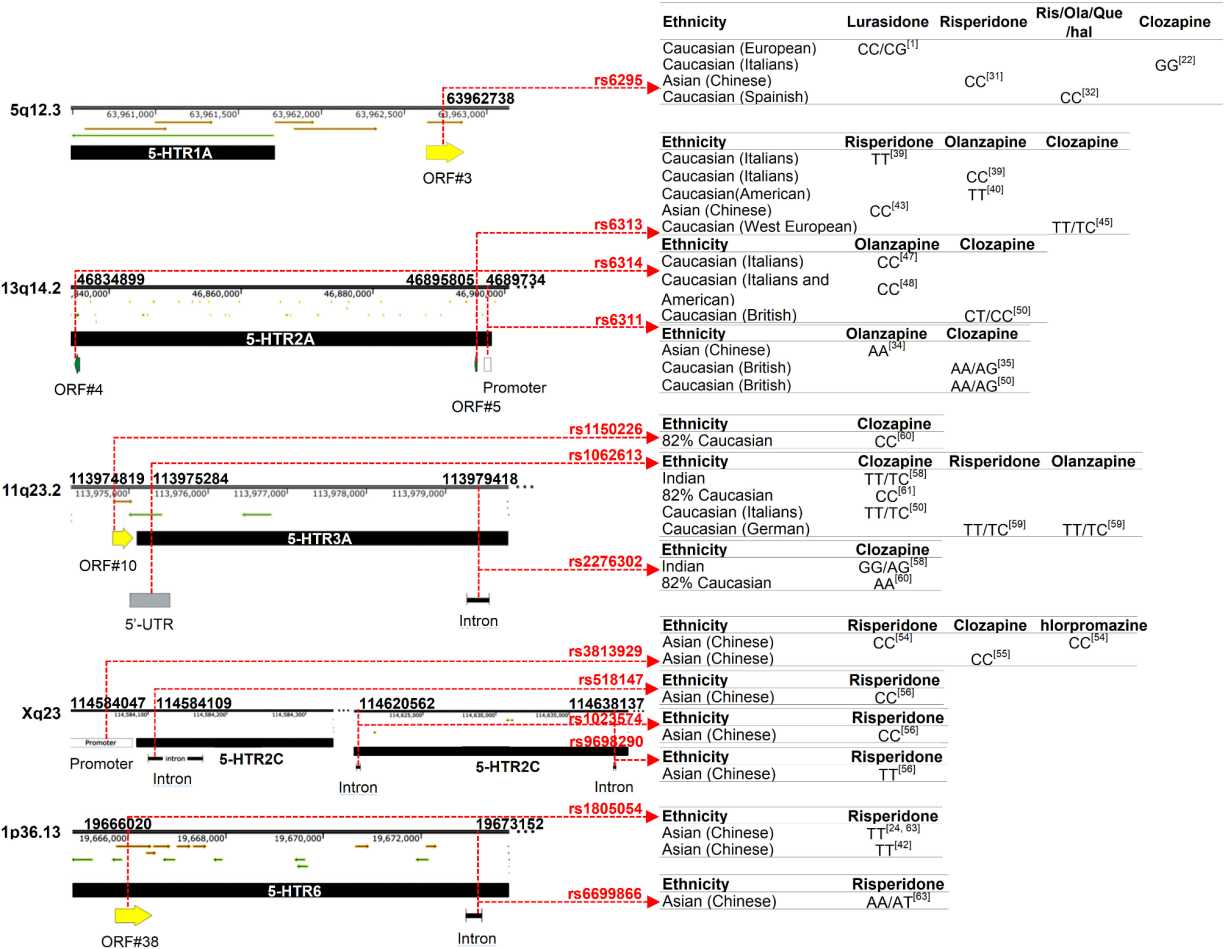
Effective genotypes of drug treatment at different SNP loci

There is a notable correlation between SNPs of the *5-HTR* gene and the efficacy of antipsychotics. This study reviews the association between SNPs in six types of *5-HTR* genes and the efficacy of atypical antipsychotics, as shown in Fig. 1:

1. The *5-HTR1A* rs6295 polymorphism locus shows varying effective genotypes for different drugs across various populations. Specifically, lurasidone treatment was more effective in patients with the CC or CG genotypes in the European Caucasian population. Meanwhile, clozapine treatment was more effective in patients with the GG genotype in the Italian Caucasian population. In contrast, risperidone treatment was more effective in patients with the CC genotype in the Chinese Asian population. Additionally, in a Spanish Caucasian population, treatment with multiple drugs (risperidone, olanzapine, quetiapine, and haloperidol) showed better efficacy in patients with the CC genotype.
2. The effective genotypes of different drugs at the *5-HTR2A* rs6313 polymorphic locus varied among different populations. In the Italian Caucasian population, patients with the TT genotype responded better to risperidone treatment, while patients with the CC genotype responded better to olanzapine treatment. In the US Caucasian population, both TT and CC genotypes showed a positive response to olanzapine treatment, whereas risperidone treatment was more effective in patients with the CC genotype. Meanwhile, in the Chinese Asian population, patients with the CC genotype responded better to risperidone treatment, while in the Western European Caucasian population, patients with the TT or TC genotype had a better response to clozapine treatment. Furthermore, patients carrying the C allele of the rs6314 polymorphic locus had a better response to atypical antipsychotics. Furthermore, in both the Italian and US Caucasian populations, olanzapine was more effective in patients with the CC genotype, while in the UK Caucasian population, clozapine showed better efficacy in patients with the CT and CC genotypes. Moreover, patients carrying allele A of the rs6311 polymorphic locus had a better response to atypical antipsychotics: In the Chinese Asian population, olanzapine treatment was more effective for patients with the AA genotype, while in the British Caucasian population, clozapine treatment was more effective for patients with the AA or AG genotype.
3. The *5-HTR3A* rs1150226 polymorphic locus showed better efficacy for clozapine treatment in patients with the CC genotype across most Caucasian populations. Most studies have indicated that the TT or TC genotypes at the rs1062613 polymorphic locus are associated with the most effective response to atypical antipsychotic treatment. For instance, in the Indian population, clozapine treatment was more effective in patients with TT or TC genotypes, while in the German Caucasian population, both risperidone and olanzapine treatment showed better efficacy for patients with TT or TC genotypes. However, some studies have also concluded that clozapine treatment is more effective in patients with the CC genotype in the Caucasian population. Additionally, the rs2276302 polymorphism showed better efficacy in the Indian population with the GG or AG genotype and in the Caucasian population with the AA genotype.
4. The rs3813929 polymorphic locus of the *5-HTR2C* gene is associated with improved treatment responses to risperidone, clozapine, and chlorpromazine in Chinese Asian patients with the CC genotype. Similarly, patients with the CC genotype in the Chinese Asian population also benefit from treatment with risperidone when they have the rs518147 and rs1023574 polymorphic loci. In contrast, the rs9698290 polymorphic locus was associated with improved efficacy in patients with the TT genotype.
5. In a Chinese Asian population, the rs1805054 polymorphic locus of the *5-HTR6* gene was associated with risperidone treatment efficacy in patients with the TT genotype, while the rs6699866 polymorphic locus was associated with efficacy in patients with either the AA or AT genotype.

Differences in the results of various studies suggest that the complexity of the causes of psychiatric disorders and the evaluation of drug effectiveness may involve unknown genetic heterogeneity, even within the same genotype group in a population. Insufficient sample sizes and short observation periods in some studies undermine the strength of the analysis results, which may also be influenced by the location of polymorphic loci. Notably, polymorphic loci in the promoter region, such as rs6311 and rs3813929, exhibit relatively small differences, while polymorphic loci in the intron region, such as rs2276302, demonstrate larger differences.

Atypical antipsychotics can cause side effects in clinical use, and certain 5-HTR polymorphic loci are associated with these side effects. However, the results are contradictory. For instance, the T allele of the rs3813929 polymorphic locus of *5-HTR2C* has been shown to protect against weight gain in patients treated with atypical antipsychotics such as olanzapine [67–69], risperidone [70], and clozapine [71, 72], while the C allele is associated with antipsychotic-induced weight gain [73]. Conversely, other studies have found no significant association between the rs3813929 polymorphism and weight gain or metabolic syndromes [69, 74–76]. Moreover, the rs3813929 polymorphism may be associated with other complications in patients with schizophrenia on medication. For example, a statistically significant association has been found between insulin levels and T allele carriers in Romanian paediatric patients with schizophrenia or bipolar disorder. Atypical antipsychotics are also linked to elevated insulin levels and insulin resistance, and the rs3813929 polymorphism can serve as a diagnostic marker for antipsychotic-induced insulin resistance [77]. Additionally, in American Caucasian patients treated with risperidone, the *5-HTR2A* rs6313 polymorphism was associated with body mass index, and there were differences in the genotype distribution of the *5-HTR2C* rs3813929 and *5-HTR2A* rs6314 polymorphisms between patients with and without extrapyramidal side effects [78]. Furthermore, the association between side effects of atypical antipsychotics and *5-HTR* gene SNPs is also a major focus in current schizophrenia research.

Haplotype information is an important database for studying pathogenic genes and conducting linkage and association analyses. Chen et al. found that haplotypes consisting of the A−1438G and T102C polymorphisms of the *5-HTR2A* gene predicted negative symptom performance upon aripiprazole treatment in Han Chinese patients with schizophrenia, and the GG/CC genotype group specifically predicted a poor aripiprazole response [8]. In contrast, one study found that haplotypes at the rs6318 locus Ser23 and the rs3813929 locus C alleles of the *5-HTR2C* gene were significantly associated with improved positive and negative symptoms in American men with schizophrenia [79]. Thus, haplotypes comprising multiple polymorphic loci may be associated with atypical antipsychotic efficacy. However, current studies have only independently analysed the association between certain *5-HTR* gene SNPs and antipsychotic drug efficacy, and no haplotypes involving different *5-HTR* genes have been identified. This could be a topic for future research. Additionally, the effective genotypes of SNPs treated with antipsychotics are not consistent among different ethnic groups, which could be another area of interest for future studies.

## Conclusions

With the rapid development of medical technology, antipsychotics are constantly being updated to improve their efficacy and safety. However, due to the complexity of patients’ genetic polymorphisms and psychiatric disorders, there are still individual differences in how effective they are. Specifically, *5-HTR* gene SNPs have been found to be associated with antipsychotic efficacy. In this paper, we reviewed the typing of the *5-HTR* gene, the effects of commonly used clinical atypical antipsychotics, and the influence of *5-HTR* gene SNPs on antipsychotic efficacy. Our goal was to provide a reference for individualised clinical treatment of schizophrenia. However, to ensure the reliability of our analysis results, further research is needed with a larger subject population, a longer observation period, more scientific drug response typing criteria, an ideal range of genetic variant detection, and more complex genotype group analysis.

## Data Availability

Not applicable.
